# Electro-acupuncture for central obesity: a patient-assessor blinded, randomized sham-controlled clinical trial

**DOI:** 10.1186/s12906-024-04340-5

**Published:** 2024-01-29

**Authors:** Tsz Fung Lam, Zipan Lyu, Xingyao Wu, Yi Ping Wong, Peihua Cao, Emily Yen Wong, Hung Bun Hung, Shiping Zhang, Zhaoxiang Bian, Linda L. D. Zhong

**Affiliations:** 1 School of Chinese Medicine, Hong Kong Baptist University, 7 Baptist University Road, Kowloon Tong, Hong Kong S.A.R, China; 2 Clinical Research Center, Zhujiang Hospital, Southern Medical University, Guangzhou, China; 3 Department of Family Medicine and Primary Care, LKS Faculty of Medicine, the University of Hong Kong, Hong Kong S.A.R, China; 4 Promed Chinese Medicine Specialist Clinic, Hong Kong S.A.R, China; 5 School of Biological Sciences, Nanyang Technological University, 60 Nanyang Drive, Singapore, 637551 Singapore

**Keywords:** Central obesity, Acupuncture, Electro-acupuncture, Randomized controlled trial

## Abstract

**Background:**

Central obesity is considered as a significant health threat to individuals. Scientific research has demonstrated that intra-abdominal fat accumulation is associated with higher metabolic and cardiovascular disease risks independent of Body Mass Index (BMI). This study aimed to evaluate the efficacy and safety of electro-acupuncture in treating central obesity compared with sham acupuncture.

**Method:**

This was a patient-assessor blinded, randomized, sham-controlled clinical trial. One hundred sixty eight participants aged between 18 and 65 years old with BMI ≥ 25 kg/m^2^ and waist circumference (WC) of men ≥ 90 cm / women ≥ 80 cm were enrolled and allocated to the acupuncture or sham acupuncture group equally. For the acupuncture group, disposable acupuncture needles were inserted into eight body acupoints, including Tianshu (ST-25), Daheng (SP-15), Daimai (GB-26), Qihai (CV-6), Zhongwan (CV-12), Zusanli (ST-36), Fenglong (ST-40), and Sanyinjiao (SP-6) with electrical stimulation. For the control group, Streitberger’s non-invasive acupuncture needles were utilized at the same acupoints with identical stimulation modalities. The treatment duration was 8 weeks with 2 sessions per week and the follow-up period was 8 weeks. The primary outcome was the change in WC before and after the treatment. The secondary outcomes were the changes in hip circumference, waist-to-hip circumference ratio, BMI, and body fat percentage during the treatment and follow-up period.

**Results:**

The acupuncture group displayed a significant change in WC compared to the sham group both treatment and follow-up period (MD = -1.1 cm, 95% CI = -2.8 to 4.1). Significant change in body fat percentage was recorded for both groups after treatment but no significance was observed during the follow-up period (MD = -0.1%, 95% CI = -1.9 to 2.2). The changes in hip circumference were also significant both treatment and follow-up period for the acupuncture group (MD = -2.0 cm, *95% CI* = -3.7 to -1.7). Compared with sham acupuncture, the body weight (MD = -1 kg, *95% CI* = -3.3 to 5.3*)*, BMI (MD = -0.5, *95% CI* = -0.7 to 1.9) also decreased significantly within and between groups. The incidence of adverse events was similar in the two groups.

**Conclusion:**

This study provided evidence that electro-acupuncture could be effective in treating central obesity by reducing WC, hip circumference, body weight, BMI, and waist-to-hip circumference ratio.

**Trial registration:**

ClinicalTrials.gov Identifier: NCT03815253, Registered 24 Jan 2019.

## Introduction

In 2020, over 2.6 billion people are considered as overweight or obese (BMI ≥ 25 kg/m^2^) globally, representing 38% of the world’s population. Among them, 14% are with BMI ≥ 30 kg/m^2^ [1]. In Hong Kong, according to the Department of Health Population Health Survey (PHS) 2020–22, 32.6% of the adult population were overweight or obese (BMI ≥ 25.0 kg/m^2^) [2]. In China, the number of people considered overweight and obesity have reached 100 million and 15 million respectively [3]. Among well-developed regions, survey reported that up to 29.9% of the people aged 15–84 were obese. As obesity is a chronic condition that can cause multiple metabolic diseases, obesity problem will cause both economic and medical burdens in the long run [4–6].

Obesity can be categorized into generalized and abdominal obesity (also known as central obesity). Central obesity is defined as the accumulation of excessive fat in the abdomen compared with the lower extremities and hips [7–9]. Apart from the commonly known correlation with type 2 diabetes, dyslipidaemia, hypertension, and abnormalities in blood coagulation and fibrinolysis, central obesity also significantly correlates with cardiovascular and cancer mortality [10–13]. In primary healthcare settings, health providers always need to handle obese patients with related chronic symptoms and diseases [14]. Moreover, patients might raise inquiries regarding various weight loss interventions including the potential risks and benefits of pharmaceutical medications [15–17].

Changes of lifestyle through diet control and physical exercise are well-known and effective solution for weight loss. However, it requires strong self-discipline for months to years [18, 19]. Meanwhile, although various types of slimming pharmaceutical products claim their effectiveness, there may be adverse effects or even rebound after the patient stop using the products [20].

Traditional Chinese medicine (TCM) provides alternative approaches towards weight control. Based on previous clinical studies and experience, acupuncture is the most acceptable TCM therapy in treating overweight and obesity [21, 22]. The effectiveness of acupuncture can be seen in improvement in fat decomposition as well as reduction of blood triglycerides levels and WC [20, 21]. Through meta-analysis and clinical studies, the related evidence of the effectiveness of weight control by acupuncture keeps on growing [23]. In this study, our objective is to provide solid evidence to support the effectiveness of electro-acupuncture in treating central obesity.

## Method

### Study design

It was a patient-assessor blinded, randomized, sham-controlled clinical trial on electro-acupuncture for central obesity. Registered TCM practitioners with at least three years of clinical experience were trained to treat participants following the study protocols. The TCM practitioners were aware of the grouping of each participant, but the participants and assessor were blinded to the group allocation.

### Sample size

The results of our pilot study with 72 participants showed that electro-acupuncture combined with auricular acupressure could reduce WC by 1.57% (SD = 0.025) from the baseline at week 8, compared with 0.14% (SD = 0.041) in the sham group [24]. Considering 80% efficacy and 5% alpha (two tails), at least 70 subjects were required in each group to test its effectiveness. Considering a 20% dropout rate, we planned to recruit 84 subjects for each group, i.e. a total of 168 subjects. Calculations were performed using PASS 11 software in Caseville, Utah, USA.

**Fig. 1:**
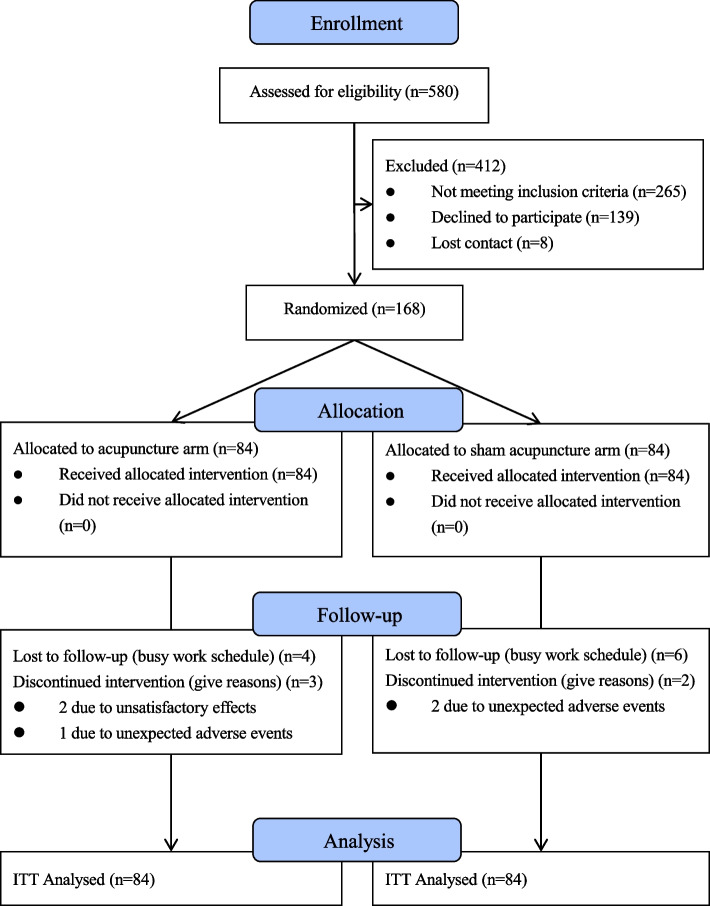
Participant Flow Diagram. A total of 580 participants underwent initial screening for eligibility. Among them, 412 participants were excluded based on predefined criteria. The remaining 168 eligible participants were randomly assigned to either the treatment group (*n* = 84), receiving electro-acupuncture, or the control group (*n* = 84), receiving sham acupuncture. Both groups underwent twice-weekly treatments over eight weeks, followed by a post-treatment follow-up session at eight weeks. Seven participants discontinued intervention or were lost to follow-up in the treatment group, and eight participants in the control group faced similar circumstances. The final analysis included 168 participants, adhering to the intention-to-treat (ITT) principle, for efficacy and safety assessments

One hundred sixty-eight participants with central obesity were recruited from the public through advertisement. Eligible participants were randomly assigned into the two groups with 1:1 ratio. The treatment group (*n* = 84) received electro-acupuncture. The control group received sham acupuncture (*n* = 84). Appropriate acupuncture frequency was the premise of effective acupuncture [25]. The frequency of acupuncture in western countries was once a week, while that in China was 2–3 times a week [26]. However, frequent clinic visits might lead to commuting concerns and compliance may be compromised, which may in turn lead to high dropout rate. Therefore, in this trial, participants were treated twice a week for a total of eight weeks, with a follow-up session scheduled eight weeks after completion of treatment. Every participant was administered 16 sessions of acupuncture in total (Fig. 1).

The primary outcome was the change in WC at the beginning and at the end of the study. Secondary outcomes included the changes in hip circumference (HC), waist-to-hip circumference ratio, BMI, body fat percentage, and body weight. All outcomes were evaluated at the 1st, 4th, 8th, 16th sessions of treatment and at the follow-up session.

### Recruitment

We recruited participants through advertisements and TV programs. Eligible participants signed the consent form before randomization.

### Participants

A total of 168 participants were screened from 580 respondents by applying the following criterion.

#### Inclusion criteria

Respondents were included in the trial if they met the following criteria:

1. In the past three months, they have not received weight-loss treatment by Chinese medicine, conventional medicine, or nutritionist;
2. Aged between 18 and 65 years old;
3. Central obesity, i.e. WC of men ≥ 90 cm or women ≥ 80 cm;
4. BMI ≥ 25 kg/m^2^.

#### Exclusion criteria

Respondents were excluded from the trial if they met the following criteria:

1. Endocrine system diseases, e.g. thyroid disorder, pituitary disorder, and sex gland disorder, etc.;
2. Impaired hepatic or renal function;
3. Heart disease, e.g. arrhythmia, heart failure, myocardial infarction, and persons implanted with a pacemaker, etc.;
4. Pregnant or lactating women;
5. Bleeding tendency;
6. Allergy and immunology disease;
7. Bleeding coagulation disorders;
8. Stroke or otherwise unable to exercise.

### Setting

The trial was conducted in four Chinese Medicine Clinics under the School of Chinese Medicine, Hong Kong Baptist University listed as below:

- Hong Kong Baptist University Mr. & Mrs. Chan Hon Yin Chinese Medicine Specialty Clinic and Good Clinical Practice Center;
- Hong Kong Baptist University Chinese Medicine Specialty Center;
- Hong Kong Baptist University ‒ Jockey Club Chinese Medicine Disease Prevention and Health Management Center; and
- Haven of Hope – Hong Kong Baptist University Chinese Medicine Specialty Clinic.

The study was approved by the Committee on the Use of Human and Animal Subjects in Teaching and Research, Hong Kong Baptist University (HASC/HASC/17–18/C03). Informed consent was obtained from all participants and the study was carried out in accordance with the Declaration of Helsinki. The recruitment started in February 2019 and the study completed in December 2020.

We designed a diet diary for participants to record their food intake and exercise patterns daily. The diary was reviewed by researchers in every treatment session and at the follow-up session.

## Interventions

### Electro-acupuncture treatment

#### The acupuncture prescription

According to a systematic review [19], Zusanli(ST-36), Sanyinjiao(SP-6), Tianshu(ST-25), Fenglong(ST-40), Zhongwan(CV-12), Qihai(CV-6) were frequently used acupuncture points in body weight control trials. In addition to these six acupuncture points, we added two acupoints on the abdomen, i.e. Daheng(SP-15) and Daimai(GB-26), that are effective for treating central obesity based on our clinical experience. There were 8 acupoints and 14 needling points in total (Table 1).

**Table 1:** Acupoints and its locations, symptoms, and indications

Acupoint	Locations	Symptoms and indications
Tianshu(ST-25)	2-inch lateral to the level with the umbilicus (CV-8)	Gastrointestinal disorders: e.g. nausea, vomiting, diarrhea Fluid metabolism disorders: e.g. excessive intake of drinks, polyuria, tumescence
Daheng(SP-15)	4-inch lateral to the center of the umbilicus (CV-8) lateral to rectus abdominus	Gastrointestinal disorders: e.g. diarrhea, constipation
Daimai(GB-26)	Directly below LV-13 at the crossing point of a vertical line through the free end of the 11th rib and a horizontal line through the umbilicus (level with CV-8)	Gastrointestinal disorders: e.g. bloating, constipation, diarrhea Gynecological disorders: e.g. dysmenorrhea, abnormal vaginal discharge
Qihai(CV-6)	Midway between CV-5 and CV-7, 1.5-inch below CV-8 (umbilicus)	Gastrointestinal disorders: e.g. abdominal pain, constipation Reproductive system disorders: e.g. irregular menstruation, infertility General weakness of the body
Zhongwan(CV-12)	Midway between CV-8 and CV-16, 4-inch above CV-8 (umbilicus)	Gastrointestinal disorders: e.g. gastric pain, vomiting, indigestion, loss of appetite
Zusanli(ST-36)	3-inch below ST-35, one finger width lateral from the anterior border of the tibia	Gastrointestinal disorders: e.g. indigestion, abdominal pain Local symptoms: e.g. lower limb pain, paralysis
Fenglong(ST-40)	8-inch below ST-35, one finger width lateral to ST-38, two finger widths lateral to the anterior border of the tibia	Fluid metabolism disorders: e.g. tumescent limbs and abdomen, sparing urine Local symptoms of the lower limbs
Sanyinjiao(SP-6)	3-inch directly above the tip of the medial malleolus on the posterior border of the tibia	Gastrointestinal disorders: e.g. nausea, diarrhea, colic Renal/reproductive system disorders: e.g. menstrual disorder, impotence, edema

The course of treatment of this clinical experiment was 8 weeks, with 2 acupuncture treatment sessions per week, making a total of 16 sessions. The needle retention time was 30 min in each session. At the beginning of the session, the TCM physician instructed the participant to lie supine on the treatment bed, exposing the abdomen and legs for disinfection before acupuncture was administered.

#### Electro-acupuncture (for the experimental group)

The TCM physician used acupuncture needles (verum acupuncture needles Asia-med Special No. 16 with 0.30 × 0.30 mm matching the Streitberger sham-needles) to puncture 8 acupoints with a total of 14 needling points. The needles were inserted through a rubber ring base to the body, which matched the sham needling procedure of Streitberger sham-needles. The insertion depth of each acupoint was about 10–25 mm to achieve Deqi sensation, a feeling of soreness, numbness, heaviness, and pressure soreness by the participant or a feeling of heavy, tightness, astringency and stagnation by the TCM physician [27]. Electrical stimulation was then applied to the abdominal points with 50 Hz densely dispersed waves at 50 V through an electric needle stimulator (ES-160 6-Channel Programmable Electro-acupuncture). The handle of the needles would start to tremble slightly after the electrical stimulation was applied. The needles would then remain for 30 min.

#### Sham acupuncture (for the control group)

Streitberger's non-invasive acupuncture needles (specification 8 × 1.2 inches / 0.30 × 30 mm) were used at the same 8 acupuncture points in the same stimulation manner, but the needles were only adhered to the skin with a rubber ring base and not inserted. The validity and credibility of the model have been fully proven. The needles were also connected to an electric needle stimulator for 30 min, but no electrical stimulation was applied. The stimulator only emits the same beeping sound and flashing light continuously.

### Lifestyle intervention

All participants were advised to follow the guidelines of Balanced Diet Food Pyramid Designed by Hong Kong Dietitian’s Association in their daily diets. Subjects were not required to do exercises, but they can continue with their prevailing exercise routine, if any.

### Outcome measures

We measured participants’ WC (i.e. the primary outcome), hip circumference, waist-to-hip circumference ratio, BMI and body fat percentage (i.e. secondary outcomes) at the 1st, 4th, 8th and 16th treatment sessions and at the follow-up session. Bodyweight, BMI, and body fat percentage were measured by body Omron Karada Scan HBF-701. Adverse events of acupuncture treatment were recorded using the Treatment Emergent Symptom Scale (TESS) and reported based on the participants' reports.

### Randomization assignment

Participants were randomly assigned to receive acupuncture (body electro-acupuncture) or control (sham). For randomization, simple, complete, non-sequential random numbers were generated in advance by a computer program in a block of four, and kept by the Principal Investigator (PI, LDZ). After confirming that the participant met all the selection criterion, the PI provided the acupuncturist with a random number corresponding to the group assignment. This design was to ensure that clinical assessors and participants were not informed of the distribution.

### Blinding process

This was a patient-assessor blinded sham-controlled clinical trial. The participants were not informed of their group assignment. Participants in the sham-controlled group received treatment with Streitberger's non-invasive acupuncture needles (specification 8 × 1.2 inches / 0.30 × 30 mm) at the same 8 acupuncture points in the same stimulation manner as the experimental group. After the treatment, the participants were asked about perceived treatment allocation to evaluate the success rate of blinding. Only the acupuncturists knew the group allocation of each participant. The clinical assessors and the statistician performing the data analyses were blinded to the group allocation throughout the study. Only when the lead investigator (PI, LDZ) considered if any parameters of a case was critical to patient safety, such as in medical emergencies will blindness be eliminated on a case-by-case basis.

### Data processing and analysis

Statistical analysis was performed using the Social Science Statistics Package (SPSS) for Windows version 23.0. Statistical significance was defined as a two-sided *P* value of < 0.05. Efficacy and safety analyses were performed by the intention-to-treat (ITT) principle. Imputation methods for dropout participants data were estimated using the last observation carried-forward method. Baseline characteristics were reported as mean (SD). Normally distributed continuous variables were evaluated using the Student's t-test, and non-normal distribution was evaluated using the non-parametric Mann–Whitney U test to assess baseline differences between the two groups. For categorical variables, chi-square tests or Fisher's precise tests were used. The analysis of covariance based on ANCOVA was used. ANCOVA was used to compare the treatment groups and subscales, with the treatment group as the model factor and the baseline as the covariate. Changes in covariate scores from baseline to the end of treatment were examined by repeated analysis of variance (ANOVA). Paired t-test was used to evaluate normally distributed data, and the Wilcoxon positive and negative rank test was used to evaluate the normal distribution data.

## Results

Of 580 respondents, 168 eligible patients were randomized to the electro-acupuncture group (*n* = 84) and sham group (*n* = 84). 91.7% (77/84) of participants in the electro-acupuncture group and 90.5% (76/84) in the sham group finished all the treatment and follow–up sessions.

The baseline characteristics of the two groups were balanced *(P* > *0.05)*. The figures were summarized in Table 2.

**Table 2:** Baseline characteristics

Characteristic	All Participants	Group A (Acupuncture)	Group B (Sham)
	**[*N* = 168]**	**[*N* = 84]**	**[*N* = 84]**
Female, *n (%)*	109 (64.9%)	59 (35.1%)	50 (29.8%)
Male, *n (%)*	59 (35.1%)	25 (14.9%)	34 (20.2%)
Mean Age (SD)	47.5 (11.0)	46.8 (11.1)	48.2 (10.9)
Mean Weight (SD), *kg*	78.7 (13.3)	79.2 (13.3)	78.2 (13.3)
Mean BMI (SD), *kg/m*^*2*^	29.7 (4.0)	29.9 (4.2)	29.5 (3.9)
Mean DBP (SD), *mmHg*	83.8 (10.2)	83.8 (10.0)	83.9 (10.6)
Mean SBP (SD), *mmHg*	127.1 (12.7)	129.5(14.0)	124.7(11.0)
Mean HR (SD) *per min*	76.6 (11.7)	75.6 (11.1)	77.5 (12.3)
Mean WC (SD) *cm*	99.1 (9.9)	98.9 (10.2)	99.2 (9.6)
Mean HC (SD) *cm*	108.0 (8.1)	108.3 (8.6)	107.7 (7.5)
Mean BF (SD) *%*	36.5 (6.3)	36.2 (6.0)	36.8 (6.7)

*BMI* Body mass index, *DBP* Diastolic blood pressure, *SBP* Systolic blood pressure, *WC* Waist circumference, *HC* Hip circumference, *BF* Body fat percentage

There was no significant difference on the baseline data between the two groups *(P* > *0.05)*

### Primary outcome

After 8 weeks of treatment, the WC of participants in the electro-acupuncture group showed a significant decrease compared with the baseline, with decrease of 1.4 cm (95% CI = -1.3 to -0.5) at week 4 and 1.8 cm (95% CI = -2.3 to -0.4) at week 8. The control group showed a decrease of 0.4 cm (95% CI = -2.2 to -0.5) in WC at week 4 and decrease of 0.6 cm (95% CI = -2.5 to -1.7) at week 8. The mean difference of the two groups was -1.0 cm (95% CI = -2.5 to 3.9) at week 4 and -1.2 cm (95% CI = -2.4 to 4.3) at week 8 (Table 3).

**Table 3:** Primary outcome within and between different groups

	**Group A (electro-acupuncture)**		**Group B (sham acupuncture)**		**Group A vs Group B**	
	**Mean Changes from Baseline (95% CI)**	***P* Value**	**Mean Changes from Baseline (95% CI)**	***P* Value**	**Differences (95% CI)**	***P* Value**
**Waist Circumference (cm)**						
Before treatment 0 wk	NA	NA	NA	NA	-0.3 (-3.4 to 2.8)	0.858
During treatment 4 wk	-1.4 (-1.3 to 0.5)	0.002	-0.4 (-2.2 to -0.5)	0.406	-1.0 (-2.5 to 3.9)	0.063
After treatment 8 wk	-1.8 (-2.3 to -0.4)	0.008	-0.6 (-2.5 to -1.7)	0.267	-1.2 (-2.4 to 4.3)	0.003
Follow up 16 wk	-1.5 (-2.7 to -0.2)	0.021	-0.4 (-2.4 to -1.5)	0.328	-1.1 (-2.8 to 4.1)	0.029

### Secondary outcomes

#### Changes in weight (kg)

Both groups recorded a decrease in body weight during treatment (MD = -0.9 kg, 95% CI = -3.0 to 5.3). The sham group had no difference during the follow-up (MD = -1.3 kg, 95%CI = -1.9 to -0.7).

Electro-acupuncture is found to be more effective in reducing body weight than sham acupuncture both in treatment and during follow up (Table 4). The changes of body weight in the follow up period were -2.3 kg (95% CI = -1.9 to -0.7) and -1.3 kg (95% CI = -1.9 to -0.7) for the experimental and sham group respectively.

**Table 4:** Secondary outcomes within and between different groups

Items	Group A (electro-acupuncture)		Group B (sham acupuncture)		Group A vs Group B	
	**Mean Changes from Baseline (95% CI)**	***P* Value**	**Mean Changes from Baseline (95% CI)**	***P* Value**	**Differences (95% CI)**	***P* Value**
**Weight (kg)**						
Before treatment 0 wk	NA	NA	NA	NA	1.0 (-3.1 to 5.2)	0.617
During treatment 4 wk	-1.6 (-1.0 to -0.3)	0.001	-0.7 (-1.0 to -0.4)	0.597	-0.9 (-3.0 to 5.3)	< 0.001
After treatment 8 wk	-3.0 (-1.4 to -0.5)	< 0.001	-1.0 (-1.4 to -0.6)	0.034	-2.0 (-3.1 to 5.3)	< 0.001
Follow up 16 wk	-2.3 (-2.0 to -0.7)	< 0.001	-1.3 (-1.9 to -0.7)	0.643	-1.0 (-3.3 to 5.3)	< 0.001
**Body Mass Index (kg/m**^**2**^**)**						
Before treatment 0 wk	NA	NA	NA	NA	0.5 (-0.8 to 1.7)	0.456
During treatment 4 wk	-0.4 (-0.6 to -0.2)	< 0.001	-0.2 (-0.8 to -0.2)	0.103	-0.2 (-0.7 to 1.8)	0.037
After treatment 8 wk	-0.4 (-0.6 to -0.1)	0.009	-0.2 (-0.9 to -0.3)	0.257	-0.2 (-0.6 to 2.0)	0.021
Follow up 16 wk	-0.6 (-0.9 to -0.3)	< 0.001	-0.1 (-1.0 to -0.3)	0.074	-0.5 (-0.7 to 1.9)	0.005
**Hip Circumference (cm)**						
Before treatment 0 wk	NA	NA	NA	NA	0.6 (-1.9 to 3.1)	0.626
During treatment 4 wk	-1.8 (-2.5 to -1.0)	< 0.001	-0.4 (-2.0 to 2.9)	< 0.001	-1.5 (-2.2 to -0.8)	0.723
After treatment 8 wk	-2.4 (-2.5 to -1.2)	< 0.001	-1.2 (-1.3 to 3.8)	< 0.001	-1.2 (-3.3 to -1.5)	0.041
Follow up 16 wk	-2.7 (-3.6 to -1.9)	< 0.001	-0.7 (-1.9 to 3.3)	0.601	-2.0 (-3.7 to -1.7)	< 0.001
**Waist-to-hip Circumference Ratio**						
Before treatment 0 wk	NA	NA	NA	NA	0.06 (-0.01 to 0.15)	0.549
During treatment 4 wk	-0.02 (-0.04 to 0.12)	0.020	-0.00 (-0.01 to 0.07)	0.861	0.03 (-0.02 to 0.89)	0.032
After treatment 8 wk	-0.03 (-0.01 to 0.15)	0.007	-0.01 (-0.02 to 0.15)	0.254	1.2 (-1.3 to 3.8)	0.001
Follow up 16 wk	-0.01 (-0.01 to 0.08)	0.549	-0.00 (-0.02 to 0.08)	0.714	0.7 (-1.9 to 3.3)	0.046
**Body Fat Percentage, %**						
Before treatment 0 wk	NA	NA	NA	NA	-0.7 (-2.7 to 1.4)	0.535
During treatment 4 wk	0.0 (-0.6 to 0.6)	0.919	-0.1 (-0.8 to 0.4)	0.435	0.1 (-2.0 to 2.0)	0.973
After treatment 8 wk	-0.3 (-0.9 to 0.3)	0.001	-0.2 (-1.8 to 0.1)	0.058	-0.1 (-2.0 to 2.1)	0.049
Follow up 16 wk	-0.3 (-0.9 to 0.4)	0.433	-0.2 (-1.8 to 0.1)	0.072	-0.1 (-1.9 to 2.2)	0.892

#### BMI

The changes in BMI at week 4, week 8, and week 16 (i.e. at follow-up) compared with the baseline (week 0) were: -0.4 (95% CI = -0.6 to -0.2), -0.4 (95% CI = -0.6 to -0.1), -0.6 (95% CI = -0.9 to -0.3) for the electro-acupuncture group; and -0.2 (95% CI = -0.8 to -0.2), -0.2 (95% CI = -0.9 to -0.3), -0.1 (95% CI = -1.0 to -0.3) for the sham acupuncture group. Electro-acupuncture was more effective than sham acupuncture both in treatment and during follow up concerning the decrease in BMI (Table 4).

#### Changes in hip circumference (cm)

The hip circumference of the electro-acupuncture group showed a significant decrease compared with the baseline. There was a significant statistical difference between the two groups. The drop in hip circumference in the electro-acupuncture group sustained to week 16 (MD = -2.7 cm, 95% CI = -3.6 to -1.9). There was also a significant statistical difference between the two groups at week 16 (MD = -2.0 cm, 95% CI = -3.7 to -1.7) (Table 4).

#### Changes of waist-to-hip circumference ratio

The change in waist-to-hip circumference ratio was summarized in Table 4. Compared with the sham group, the electro-acupuncture group showed better improvement in the waist-to-hip circumference ratio after treatment (MD = 1.2, 95% CI = -1.3 to 3.8)and follow-up (MD = 0.7, 95% CI = -1.9 to 3.3). The sham acupuncture group did not show any significant changes for this index.

#### Changes of body fat percentage

The change of body fat percentage of both the electro-acupuncture group and sham group was listed in Table 4. The acupuncture group showed significant improvement in the body fat percentage after the treatment (MD = -0.3, 95% CI = -0.9 to 0.3) but there was no further improvement at week 16. The sham acupuncture group did not show any significant changes for this index.

## Safety and adverse events

Both groups were well tolerated. There were no serious adverse events (e.g. requiring hospital admission). In the electro-acupuncture group, adverse events reported were headaches (*n* = 4), dizziness (*n* = 2) and insomnia (*n* = 5). In the sham group, adverse events reported were stomachache (*n* = 1), headache (*n* = 2) and worsening of depression (*n* = 3). All adverse events were reported to be mild. One patient withdrew from the study due to headache.

## Discussion

In this study, electro-acupuncture was found to be effective in reducing WC, hip circumference, body fat percentage, body weight and BMI when compared with sham acupuncture during the treatment period*(P* < *0.05)*. It was observed that during the follow up period at week 16, the electro-acupuncture group still shown significant improvement in body weight, BMI, waist-to-hip circumference ratio. This might indicate the persistent effects of the therapy and its sustainable benefits on central obesity. The study replicated the conclusion of the effectiveness of acupuncture in weight control from our pilot study, showing consistent effects on Hong Kong people.

Acupuncture was a well-accepted therapy in China and East Asia especially for chronic disorders like obesity, pain and idiopathic constipation [21, 24, 25]. In our trial, we selected five abdominal points (Tianshu ST-25, Daheng SP-15, Daimai GB-26, Qihai CV-6, Zhongwan CV-12) and three points from stomach meridian and spleen meridian (Zusanli ST-36, Fenglong ST-40, Sanyinjiao SP-6) at the lower limbs based on evidence from a systematic review and local experts’ consensus. Among the utilized acupoints, the classical functions of the abdominal points were harmonizing gastrointestinal functions and treating localized issues, which corresponded to excessive adipose tissue deposition in the abdomen. Besides, the selected acupoints at the lower limbs were considered to be effective in stabilizing gastrointestinal functions and enhancing fluid drainage [28].

We did not analyze our data separately based on the gender of participants. But it was observed that female subjects in the electro-acupuncture group showed larger reduction in WC than male participants. Some female subjects reported improvement on their menstruation. The findings correlated to the gynecological effects of the acupoints Daimai GB-26, Qihai CV-6 and Sanyinjiao SP-6. Studies on female endocrinal-caused obesity in Polycystic ovary syndrome or Perimenopause could be developed to further investigate these results.

Increased satiation and satiety were reported by some of the subjects in the treatment group. Studies on psychological conditions such as mood-influenced satiety and the treatment of binge eating could be developed with the data from this clinical trial. Scalp points could be selected as part of the core points of such treatment. The chief effect of these points was to regulate brain functions resulting in ease of mental stress and easing symptoms of psychological disorders. Therefore, integrating scalp-abdomen-lower limb points could be suggested for a better and holistic approach to treating obesity.

Patients' diet could influence the effectiveness of the treatment. The fat metabolized by the body after electro-acupuncture could be offset by their daily calorie intake, if excessive. In such cases, the effect of the treatment might be overtaken. We suggested that obese patients to adhere to the normal daily calorie intake suitable for their gender, age, and activity level. Diets resulting in daily calorie deficit might not be necessary for better compliance by the subjects.

Central obesity was a major risk factor of diabetes, cardiovascular disease, and colorectal cancer worldwide. On the other hand, inflammation was a well-known risk factor for the initiation and perpetuation of obesity [29]. Our study provided an option without pharmaceutical treatment and severe side effects for obese patients. Moreover, the mechanism of the efficacy also needed to be further investigated. For example, acupuncture may play a role in the regulation of glucolipid metabolism. It was illustrated that electro-acupuncture could reduce the serum levels of total cholesterol, triglycerides, low-density lipoprotein, lipoprotein A and apolipoprotein B, but enhance the level of insulin and C peptide [22, 30, 31]. This could be further evaluated in our next stage research moving from clinical trial evidence to exploring the mechanism behind the findings.

The study had some limitations. It did not involve any blood tests to verify the metabolic changes, which could be essential for future exploration. We also could not analyse the age-related changes due to the small sample size and we need a more evenly distributed age of participants. Furthermore, multi-arm studies could be designed to evaluate and explore the cost-effectiveness of acupuncture therapy precisely.

In conclusion, this single-blinded, randomized controlled clinical trial provided evidence of the efficacy and safety of electro-acupuncture in treating central obesity of Hong Kong people. It also provided reference to clinical practitioners in utilizing acupuncture for treating obesity. The mechanism of how electro-acupuncture treats obesity should be evaluated in our further study.

## Data Availability

Details of this study are available from the corresponding author upon request.

## Abbreviations

BMI: Body mass index
TCM: Traditional Chinese Medicine
WC: Waist circumference
HP: Hip circumference
BF: Body fat percentage
